# Polmacoxib: A Review of the Newer Non-steroidal Anti-inflammatory Drug in Osteoarthritis

**DOI:** 10.7759/cureus.58446

**Published:** 2024-04-17

**Authors:** Raju Easwaran, Urvi K Mistry, Milind Bhole, Kartik Peethambaran

**Affiliations:** 1 Orthopaedics and Traumatology, Shree Meenakshi Orthopaedics and Sports Medicine Clinic, New Delhi, IND; 2 Established Pharmaceuticals Division, Abbott Healthcare Pvt. Ltd., Mumbai, IND

**Keywords:** polmacoxib, osteoarthritis, cox-2 inhibitor, carbonic anhydrase, western ontario and mcmaster universities arthritis index (womac)

## Abstract

Osteoarthritis represents a huge socioeconomic burden and has a significant impact on daily life and productivity. Non-steroidal anti-inflammatory drugs (NSAIDs) are widely used in the management of osteoarthritis to curb inflammation, pain, and stiffness and improve physical function. However, due to the various side effects, most healthcare professionals avoid using NSAIDs for a long period. Nonselective cyclooxygenase (COX) inhibitors and cyclooxygenase-1 (COX-1) inhibitors are associated with increased gastrointestinal adverse effects due to the inhibition of prostaglandins, which are responsible for protecting the gastric mucosa. Cyclooxygenase-2 (COX-2) inhibitors are associated with an increased incidence of adverse cardiovascular effects due to their COX-2 inhibitory activity in the circulatory system. Therefore, there is a need for a newer NSAID that has a better safety profile to be used in osteoarthritis. Polmacoxib is a new, orally active, first-in-class NSAID that is a dual inhibitor of COX-2 and carbonic anhydrase (CA). The dual mode of action exhibited by polmacoxib is expected to minimize adverse cardiovascular effects while achieving maximum effectiveness in inflamed osteoarthritic joints. This article aims to review the pharmacological properties, clinical efficacy, and safety data of polmacoxib in osteoarthritis.

## Introduction and background

Burden of osteoarthritis

The most common chronic rheumatic disease and one of the foremost causes of pain and disability worldwide is osteoarthritis. It is the fourth most common cause of disability, and it primarily affects the hips or knees [1]. In 1990, around 23.46 million people in India suffered from osteoarthritis; in 2019, the number rose to 62.35 million [2]. Osteoarthritis is associated with a variety of modifiable and nonmodifiable risk factors, including obesity, inactivity, genetics, bone density, occupational injury, trauma, aging, and female gender [3]. Age is an important predisposing factor for osteoarthritis [1]. In Asia, the proportion of people aged 65 years and over is expected to more than double over the next two decades, rising from 6.8% in 2008 to 16.2% in 2040 [1].

Rural communities with osteoarthritis are the hardest hit due to the routine need to perform heavy physical labor coupled with inadequate access and a huge infrastructure gap with respect to treatment facilities including joint replacements. On the other hand, obesity is on the rise in developed countries, which is another common factor that favors the development of osteoarthritis [1].

Non-steroidal anti-inflammatory drugs (NSAIDs) remain the mainstay and the first line of treatment for most patients who develop disabling pain due to osteoarthritis [4,5]. This review focuses on the recently approved NSAID in India, polmacoxib. Polmacoxib is currently being marketed in South Korea and India [6-8].

Non-steroidal anti-inflammatory drugs

NSAIDs are a class of medications approved as analgesics, antipyretics, and anti-inflammatory drugs. NSAIDs exert their therapeutic effects via inhibition of cyclooxygenase (COX) enzymes. There are two COX isoenzymes, namely, COX-1 and COX-2. COX-1 is constitutively present in the body and produces prostaglandins that are responsible for protecting the gastric mucosa and activating platelets. COX-2 is inducibly expressed in the body during an inflammatory process and produces prostaglandins that are responsible for inflammation, fever, and pain [4].

Most of the available NSAIDs are nonselective inhibitors of COX, which inhibit both COX-1 and COX-2. However, selective COX-2 inhibitors only target COX-2 and spare COX-1 inhibition, thus reducing gastrointestinal damage by enabling the production of protective prostaglandins by COX-1 and maintaining the anti-inflammatory effects by inhibiting COX-2 in inflamed tissues [4]. The Osteoarthritis Research Society International (OARSI) guidelines for the nonsurgical management of knee, hip, and polyarticular osteoarthritis recommend COX-2 inhibitors in patients with gastrointestinal comorbidities over nonselective and COX-1 inhibitors [5]. However, certain COX-2 inhibitors such as valdecoxib and rofecoxib have been withdrawn globally due to a linkage with adverse cardiovascular events caused by their COX-2 inhibitory activity in the circulatory system [9].

## Review

Polmacoxib

**Figure 1:**
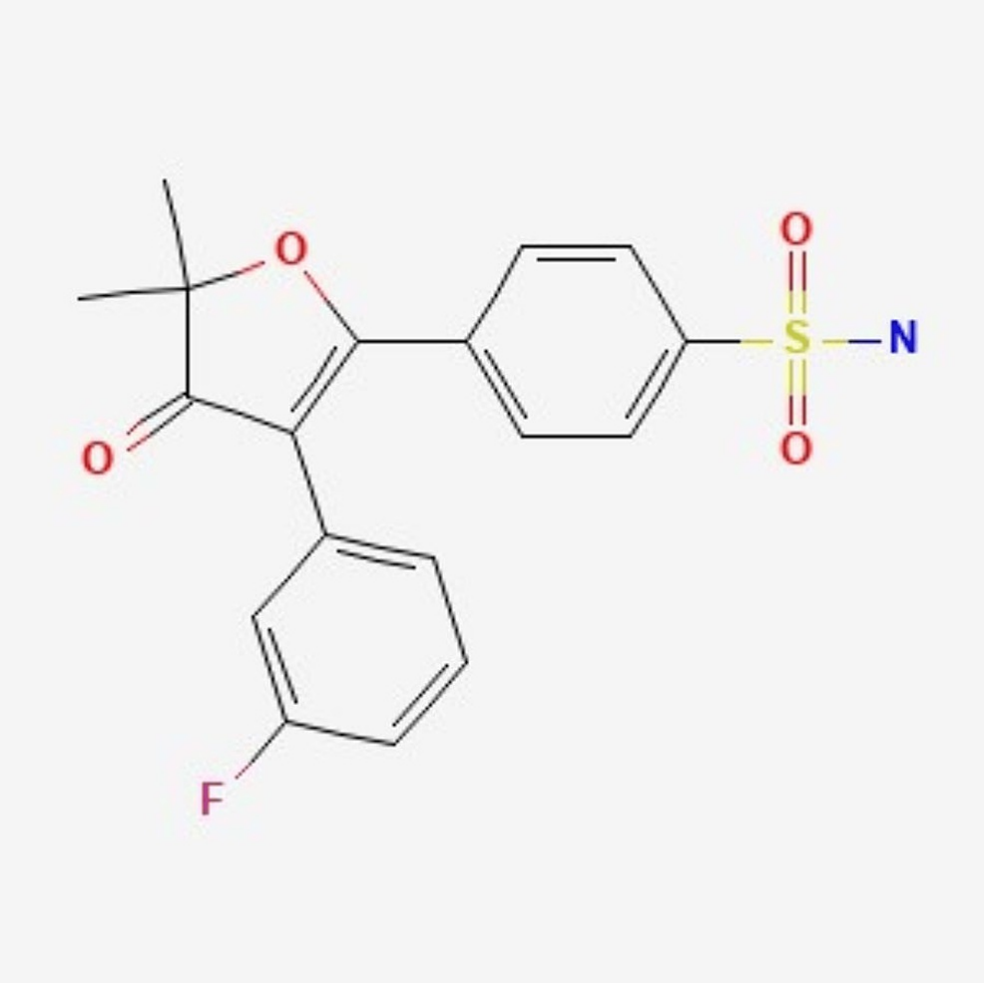
Chemical structure of polmacoxib. Source: [13].

Polmacoxib was first approved in South Korea in 2015 for the treatment of colorectal cancer and osteoarthritis. It is a first-in-class NSAID with a dual inhibitory action on COX-2 and carbonic anhydrase (CA) enzymes (Figure 1) [6-12].

Polmacoxib (2 mg) received approval from the Drug Controller General of India on February 14, 2023, for the treatment of idiopathic primary osteoarthritis of the hip and knee [8].

Pharmacodynamics of polmacoxib

Distinct from other NSAIDs, polmacoxib has a dual mechanism of action that includes inhibition of COX-2 and inhibition of CA-I/II. Most conventional COX-2 inhibitors do not show significant activity toward CA inhibition and demonstrate notable COX-2 inhibition in the cardiovascular system that could result in the development of adverse cardiac events [7].

The cardiovascular system exhibits the presence of both CA and COX-2 with an abundance of CA in the whole blood, blood vessels, and cardiovascular tissues. Where COX-2 and CA co-exist, polmacoxib demonstrates a far higher affinity to CA than COX-2, and this, in turn, reduces the COX-2 inhibitory activity in the cardiovascular tissues. The dual-action mechanism of polmacoxib can, therefore, potentially help minimize the adverse cardiovascular effects of COX-2 inhibition. Additionally, it has been noted that there is a negligible effect on the overall CA functioning of the circulatory system by the low-dose administration of polmacoxib. Conversely, inflamed tissues are deficient in CA, but express increased COX-2 due to the presence of inflammatory processes. Synovial fluid has been found to contain negligible CA. As a result, polmacoxib fully inhibits COX-2 in the inflamed joint tissues, thus alleviating inflammation and pain associated with osteoarthritis [10].

Pharmacokinetics of polmacoxib

Erythrocytes play an important role by acting as a reservoir for polmacoxib, transporting the drug in a protected, inactive state to tissues with low CA activity, such as arthritic joints. Polmacoxib shows 85- to 100-fold higher concentrations in the whole blood, i.e., erythrocytes, than in plasma where there is no CA [12]. Erythrocytes provide a *tissue-specific* transport mechanism delivering sustained levels of the drug to CA-deficient inflamed tissues. This, in turn, helps maintain low systemic exposure as polmacoxib is transported in a combined state with CA within the erythrocytes. Thus, polmacoxib is believed to offer maximum effectiveness in inflamed osteoarthritic joints while reducing its effects on the cardiorenal system or the gastrointestinal tract [6,7,11].

Following oral administration of single doses of polmacoxib 2 and 8 mg, the observed mean (standard deviation [SD]) maximum plasma concentration (*C*_max_) values were 3.5 (0.9) and 14.1 (3.7) ng/mL, respectively, and the respective time to maximum plasma concentration (*T*_max_) values were 5.6 (1.0) and 5.0 (1.7) hours. The area under the plasma concentration-time curve (AUC) following a 2 mg single dose of polmacoxib was 632.9 (162.1) ng/mL × h, whereas the AUC following an 8 mg single dose of polmacoxib was 2,366.8 (761.9) ng/mL × h [14]. The mean elimination half-lives following single oral doses of polmacoxib 2 and 8 mg were found to be 131 (19) and 127 (33) hours, respectively [15].

Based on the pharmacokinetic profile (central elimination rate constant and redistribution rate constant), it was observed that polmacoxib has a longer residence time in inflamed osteoarthritic joints as compared with blood. From a drug tolerability perspective, this suggests that other body compartments are spared from prolonged exposure to the drug. Polmacoxib is primarily excreted via the fecal route, while small amounts are also excreted via the urinary route. Therefore, a decrease in hepatic catabolism due to any reason may likely affect the clearance of polmacoxib [11]. The pharmacological properties of polmacoxib have been summarized in Table 1.

**Table 1:** Summary of features and properties of polmacoxib 2 mg.

Parameter	Features and properties
Class	Non-steroidal anti-inflammatory drugs [7]
Therapeutic use	Osteoarthritis of the hip or knee [8]
Dosage and administration	For oral use, 2 mg once daily after a meal. The dose should not exceed 2 mg/day [8]
Pharmacodynamics	Dual mechanism of action: inhibition of COX-2 and inhibition of carbonic anhydrase (CA) with high affinity [7,10]
Pharmacokinetics	Mean (SD) *T*_max_: 5.6 (1.0) hours; mean (SD) *C*_max_: 3.5 (0.9) ng/mL; mean (SD) *t*_1/2_:131 (19) hours; excretion: primarily via the fecal route [14,15]

Clinical safety and efficacy of polmacoxib

One of the foremost studies on polmacoxib was carried out by Lee et al. in South Korea, and the study aimed to compare the efficacy and safety of polmacoxib, celecoxib, and placebo in patients with osteoarthritis. This was a six-week, phase III, randomized, double-blind, parallel-group study in which 362 patients with osteoarthritis were randomized in a 2:2:1 ratio and received either polmacoxib 2 mg, celecoxib 200 mg, or placebo once a day. For safety assessment, the treatment period of six weeks was followed by a single-arm, open-label extension phase of 18 weeks during which all participants from all three arms received polmacoxib 2 mg. The primary endpoint was change in the Western Ontario and McMaster Universities (WOMAC) pain subscale score, while secondary endpoints included the WOMAC osteoarthritis index, WOMAC stiffness and physical function subscale scores, and physicians‘ and subjects’ global assessment of study drugs. The mean least square (LS) reduction in WOMAC pain subscale score from baseline to week 6 was significantly greater in the polmacoxib and celecoxib groups compared with the placebo group. The difference in the WOMAC pain subscale score at week 6 between the polmacoxib and placebo treatment arms was -2.5 (95% confidence interval [CI] -4.4 to -0.6; *P *= 0.011) and that between the polmacoxib and celecoxib treatment arms was 0.6 (CI -0.9 to 2.2; *P* = 0.425). Similarly, for other secondary endpoints, including WOMAC osteoarthritis index, WOMAC stiffness score, and WOMAC physical function score, the LS mean changes at week 6 were significantly greater in the polmacoxib and celecoxib groups than in the placebo group, whereas there was statistically insignificant difference between the polmacoxib and celecoxib groups. Thus, polmacoxib 2 mg demonstrated superior efficacy compared to placebo and noninferiority to celecoxib 200 mg at six weeks (using noninferiority margin = 5). Physicians’ global assessment indicated that a greater proportion of subjects felt *much improved* at week 3 with polmacoxib as compared with celecoxib or placebo. The results demonstrated during the 18-week polmacoxib extension phase were uniform with those observed during the polmacoxib treatment period of six weeks. Improvements in WOMAC subscale scores from baseline were maintained through 24 weeks in the polmacoxib/polmacoxib group. Some of the adverse events (AEs) commonly reported in the polmacoxib and celecoxib arms were related to gastrointestinal and general disorders including abdominal pain, diarrhea, dyspepsia, and peripheral edema. Polmacoxib was relatively well tolerated [10].

Another randomized, double-blind study by Schmidt et al. involving 248 patients with osteoarthritis investigated the clinical efficacy and safety of different doses of polmacoxib. The study aimed to evaluate three parallel dosing regimens of polmacoxib versus placebo. Polmacoxib was administered once daily in the morning at initial loading doses (day 0) and at maintenance doses (days 1-20) of 8 mg + 1.2 mg/day (high dose), 4 mg + 0.6 mg/day (medium dose), or 2 mg + 0.3 mg/day (low dose). The polmacoxib high-dose group demonstrated more than a 2-fold improvement as compared to placebo in the primary endpoint of change in the WOMAC score from baseline to day 21 (median 37% vs. 17%, respectively; *P *= 0.01). Throughout the 35-day treatment and follow-up period, the high-dose group demonstrated clinically and statistically significant superiority over the placebo group in terms of the WOMAC osteoarthritis score (*P *= 0.010) and WOMAC subscale scores for pain (*P *= 0.016), stiffness (*P *= 0.023), and physical function (*P *= 0.010). Furthermore, polmacoxib demonstrated statistically significant improvements in weekly pain relief scores at days 7, 14, 21, and 28 (*P *< 0.05 at all periods), suggesting polmacoxib has an early onset of action and provides sustained efficacy. There were no treatment-related changes in blood pressure and no incidence of gastrointestinal bleeding or other clinically relevant adverse gastrointestinal side effects. All doses of polmacoxib were well tolerated [7].

Thus, the unique mode of action of polmacoxib is postulated to have beneficial effects in terms of achieving efficacy as well as minimizing systemic side effects associated with other conventional NSAIDs [7,10]. However, there is a lack of long-term safety studies on polmacoxib, and evidence of its cardiovascular safety needs to be assessed in the future.

## Conclusions

Polmacoxib is a novel, first-in-class, orally active NSAID that acts as a dual inhibitor of COX-2 and CA enzyme. Being a selective COX-2 inhibitor, it helps reduce gastrointestinal side effects typically caused by conventional nonselective NSAIDs. Data from clinical studies suggest that polmacoxib provides statistically and clinically significant analgesic and functional benefits. Thus, polmacoxib has the potential to be used as an effective, well-tolerated, long-term pain relief drug in the management of osteoarthritis. Furthermore, its unique, tissue-specific transport mechanism could potentially help minimize the adverse cardiovascular effects of COX-2 inhibition. However, it is imperative to study the long-term efficacy and safety of polmacoxib in more large-scale randomized clinical trials.
